# Irreversibility of recursive Heaviside memory functions: a distributional perspective on structural cognition

**DOI:** 10.1007/s11571-025-10346-7

**Published:** 2025-11-28

**Authors:** Changsoo Shin

**Affiliations:** Seoul National University, Seoul, Republic of Korea

**Keywords:** Interoception, Stress parameter, Recursive cognitive processing, Awareness dynamics, Memory encoding

## Abstract

Modern AI systems excel at pattern recognition and task execution, but they often fall short of replicating the layered, self-referential structure of human thought that unfolds over time. In this paper, we present a mathematically grounded and conceptually simple framework based on smoothed step functions—sigmoid approximations of Heaviside functions—to model the recursive development of mental activity. Each cognitive layer becomes active at a specific temporal threshold, with the abruptness or gradualness of activation governed by an impressiveness parameter $s_i$, which we interpret as a measure of emotional salience or situational impact. Small values of $s_i$ represent intense or traumatic experiences, producing sharp and impulsive responses, while large values correspond to persistent background stress, yielding slow but sustained cognitive activation. We formulate the recursive dynamics of these cognitive layers and demonstrate how they give rise to layered cognition, time-based attention, and adaptive memory reinforcement. Unlike conventional memory models, our approach captures thoughts and recall events through a recursive, impressiveness-sensitive pathway, leading to context-dependent memory traces. This recursive structure offers a new perspective on how awareness and memory evolve over time, and provides a promising foundation for designing artificial systems capable of simulating recursive, temporally grounded consciousness.

## Preamble: recursive cognitive structures and self-track modeling

Our cognitive model is based on the recursive Heaviside sequence function, which describes the layered, self-referencing, and threshold-based behavior of human thought. This mathematical framework helps explain how we become aware of events, form memories, and later recall, suppress, or reinterpret them. We define the recursive cognitive potential $u(t)$ as:

$$ u(t) = 1 - H_{N_1}\left( -t + \tau _1 H_{N_2}\left( -t + \tau _2 H_{N_3}\left( -t + \tau _3 \cdots \right) \right) \right) , $$

where:

- $\tau _i$: the cognitive threshold time at layer $i$, marking when a mental shift is triggered by stress,
- $N_i$: the sharpness parameter that controls how quickly or steeply the transition happens.

### Stress Parameter and Its Relation to Sharpness

To describe sharpness in terms of stress, we define the stress parameter $s_i$ as the inverse of $N_i$:

$$ s_i = \frac{1}{N_i}. $$

When $s_i$ is small (meaning $N_i$ is large), the mental change is sharp and hard to reverse, often caused by strong or emotional experiences. When $s_i$ is large (i.e., $N_i$ is small), the change is smoother and longer-lasting, as seen in weak or ongoing stimuli. The value of $s_i$ is not fixed. It can change depending on the person, the situation, and past experiences. It reflects how sensitive someone is to stress and how much attention they give at each cognitive layer. This dual view allows us to use $N_i$ in mathematical formulas, while using $s_i$ to interpret cognitive stress in real-world terms. We use a sigmoid function to smoothly approximate each cognitive threshold:

$$ H_{N_i}(x) = \frac{1}{1 + \exp (-2N_i x)} = \frac{1}{1 + \exp \left( -\frac{2x}{s_i}\right) }. $$

**Fig. 1:**
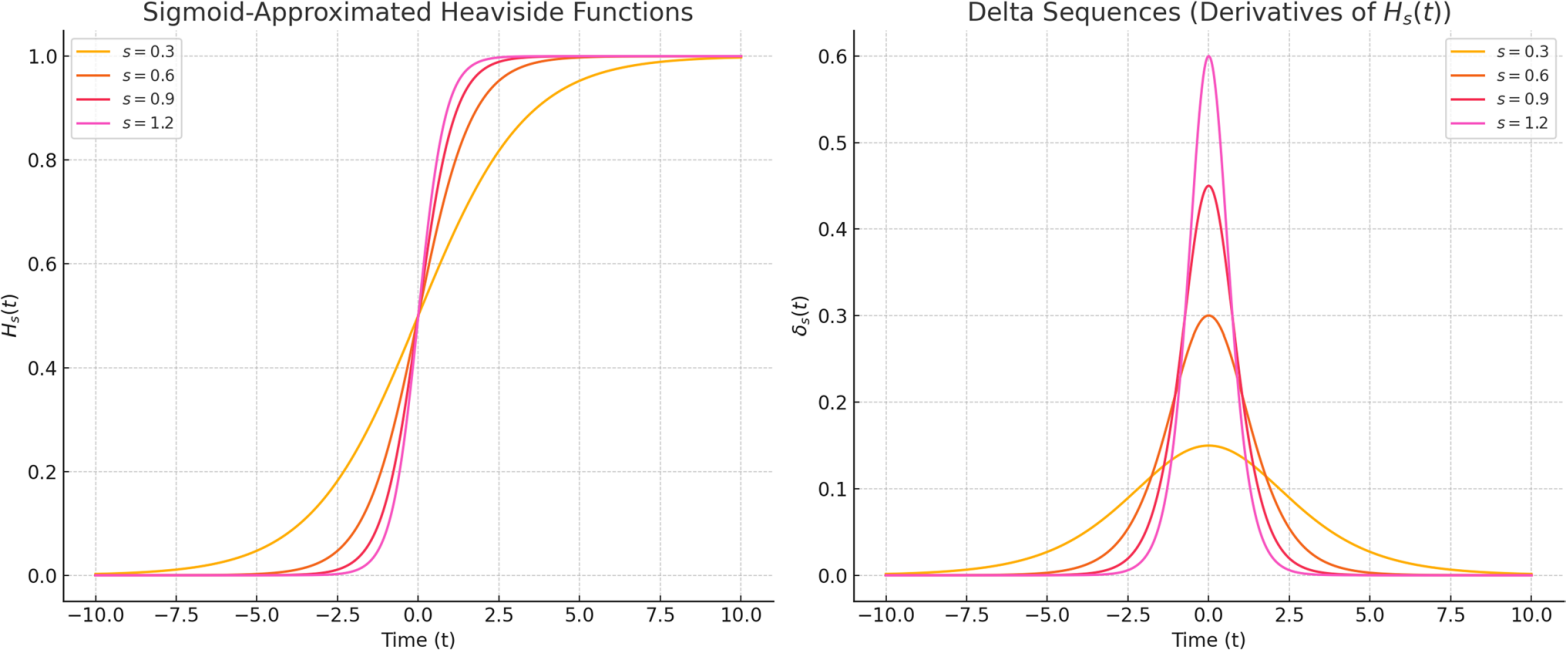
Left: Sigmoid approximations $H_{N_i}(x) = 1/(1 + \exp (-2N_i x))$, where large $N_i$ (lower stress) leads to sharper transitions. Right: Delta-like derivatives $\delta _{N_i}(t)$, which show how fast mental change occurs at each recursive layer. We define the sensitivity parameter (stress parameter/sharpness parameter/impressiveness parameter) as $s_i = 1/N_i$ to represent sensitivity to stress in cognitive processing

This function creates smooth transitions that are still nested in layers, making it easier to compute derivatives. This formulation draws from classical uses of Heaviside and delta sequences in mathematical physics (Arfken et al. 2013), where such functions are used to model sudden or layered changes in physical systems. Here, we extend that logic to cognitive structures, using recursive and smoothed versions to describe layered awareness and memory. As shown in Fig. 1, these sigmoid layers shape recursive mental activity, and their derivatives produce delta-like signals that mark key moments of awareness or memory access.

## Preamble: recursive thresholds and adaptive sharpness

At the gist of this model is the recursive Heaviside architecture, in which each cognitive layer is characterized by a threshold $\tau _i$ and a sharpness parameter $s_i$. The thresholds $\tau _i$ serve as temporal anchors that denote the onset of memory encoding or cognitive transitions, whereas the sensitivity parameters (stress parameter/sharpness parameter/impressiveness parameter) $s_i$ regulate the responsiveness of each layer—determining how abruptly the mental potential activates in the vicinity of its threshold. Together, these parameters define the temporal structure and sensitivity of recursive cognitive dynamics.

Importantly, we interpret each $s_i$ not as a fixed constant, but as a dynamic variable shaped by environmental pressures, emotional responses, and personal traits. Even under identical conditions, individuals vary in how strongly they react: what triggers intense memory in one person may pass unnoticed in another. This variability reflects how free will, temperament, and adaptation influence the cognitive sensitivity (stress/sharpness/impressiveness) $s_i$.

From the moment of birth—indeed, from the very act of being born—humans begin experiencing a series of stress-inducing thresholds. Emerging from the womb into air, sound, and separation initiates the first abrupt transition in cognition. Since then, every fundamental act—eating, sleeping, interacting—imposes layered demands on the system. Thus, existence itself is recursive stress, and cognition must adapt layer by layer.

In this view, $s_i$ functions as a mathematical reflection of both innate individuality and existential tension. It encodes how each person uniquely internalizes and responds to the unavoidable cascade of thresholds that define conscious life.

## Preamble: recursive cognitive structures and temporal directionality

The core mechanism of our model is a recursively defined Heaviside function, designed to represent the hierarchical activation thresholds that underlie cognitive processing. The cognitive potential is expressed as:

$$ u(t) = 1 - H_{s_1}\left( -t + \tau _1 H_{s_2}\left( -t + \tau _2 H_{s_3}\left( -t + \tau _3 \cdots \right) \right) \right) $$

Here:

- $\tau _i$: the memory threshold for layer $i$,
- $s_i$: sensitivity (stress/sharpness/impressiveness) of activation (related to stress or emotional strength),
- $H_{s_i}$: a smoothed Heaviside function modeling mental activation.

### No need for an external time window

 Unlike many time-series models that require a separate windowing function to delimit the past and future, this recursive structure inherently blocks activation before threshold time $\tau _i$. This is due to the use of terms like $H(-t + \tau _i)$, which vanish for $t < \tau _i$. Hence, the memory structure activates only after the corresponding cognitive event has occurred.

*This means:* the model naturally enforces time irreversibility— **no future memory acts on the past**, and no memory layer is active before its own formation. Therefore, we do not add artificial windowing functions; the recursive architecture already defines a natural causal direction.

This feature also aligns with philosophical and physical principles:

- Kant’s a priori intuition of time,
- Heidegger’s forward-thrown Dasein,
- The unidirectional flow of entropy in physics,
- and the phenomenological experience of memory and dreaming.

## Key notation and mathematical definitions

**Table:** 

Symbol	Definition and role
$t$	Global time variable (flows forward). Drives evolution of cognition. In figures, time flows right to left ($+\infty \rightarrow -\infty$) to show inward recursion.
$\tau _i$	Threshold time at layer $i$; triggered when accumulated stress activates a memory or awareness layer.
$s_i$	Sensitivity (stress/sharpness/impressiveness) parameter; inverse of sharpness. Small $s_i$: sharp, rigid response. Large $s_i$: smooth, gradual response.
$H(x)$	Standard Heaviside function: $$ H(x) = {\left\{ \begin{array}{ll} 0 & x < 0 \\ 1 & x > 0 \end{array}\right. } $$.
$H_{s_i}(x)$	Smoothed Heaviside: $H_{s_i}(x) = \dfrac{1}{1 + \exp (-2x/s_i)}$; sharpness depends on $s_i$.
$\delta (x)$	Dirac delta function: idealized impulse at $x = 0$.
$\delta _{s_i}(x)$	Smoothed delta function: $\delta _{s_i}(x) = \dfrac{2}{s_i} \cdot \dfrac{e^{-2x/s_i}}{(1 + e^{-2x/s_i})^2}$. Peaks at $x = 0$.
$u(t)$	Recursive potential: $u(t) = 1 - H_{s_1}(-t + \tau _1 H_{s_2}(-t + \tau _2 \cdots ))$. Models layered awareness/memory.
$\frac{\partial u}{\partial t}$	Time derivative of $u(t)$; represents moment-to-moment cognitive activation.
$\frac{\partial u}{\partial \tau _i}$	Threshold derivative at layer $i$; measures response sensitivity. Form: $\delta _{s_i}(-t + \tau _i) \times (\cdots )$.
$\frac{\partial u}{\partial s_i}$	Derivative w.r.t. stress sharpness: $-\frac{2(-t + \tau _i)}{s_i^2} \cdot \frac{e^{-2(-t + \tau _i)/s_i}}{\left( 1 + e^{-2(-t + \tau _i)/s_i} \right) ^2}$.
$\prod \limits _{i=1}^n \delta _{s_i}(-t + \tau _i)$	Product of delta functions; arises in higher-order resonance effects.

## Introduction

Understanding the structure of human cognition has long been a subject of philosophical and scientific inquiry. Rather than treating cognition as a passive reflection of the external world, many traditions have emphasized its active, constructive nature. This paper builds upon that premise and proposes a formal model that captures the recursive, layered dynamics of awareness and memory. We introduce a recursive Heaviside sequence function to represent how cognitive thresholds accumulate and interact over time, producing structured mental states. This mathematical formulation allows us to explore the dynamic unfolding of cognition through temporally nested activations, providing a bridge between phenomenological observations and formal models.

Recent theories in cognitive neuroscience have proposed that the brain operates through hierarchical, predictive mechanisms that continuously monitor internal states and update beliefs accordingly (Friston 2010; Seth 2013). These frameworks support the idea that self-referential, recursive processes are not only plausible but fundamental to cognition. Drawing on this perspective, our model formalizes such recursive monitoring through a nested, threshold-driven function structure.

In this paper, we introduce a mathematical model of consciousness based on a recursively layered Heaviside function, which captures the structural property of temporally nested thresholds. While our model is purely formal, it shares structural parallels with integrative frameworks such as Global Workspace Theory (GWT) (Dehaene 2014; Crick and Koch 1998; Koch 2004; Tononi 2008; Baars 1988) and related models of conscious access (Graf et al. 1984; Marcel 1983). Specifically, our recursive functions provide an abstract way to represent how temporally distributed inputs may give rise to higher-order awareness, without attempting to replicate the neural mechanisms proposed in those theories. These functions, while mathematically recursive, are intended to illustrate formal patterns of layered activation rather than detailed biological dynamics.

Artificial intelligence has also advanced memory architectures—ranging from RNNs and LSTMs (Cho et al. 2014; Elman 1990; Hochreiter and Schmidhuber 1997) to Transformer-based models (Vaswani et al. 2017), Neural Turing Machines (Graves et al. 2014; Graves et al., 2016), and memory-augmented frameworks like Retrieval-Augmented Generation (RAG) (Khandelwal et al. 2020; Lewis et al. 2020; d’Avila Garcez et al. 2009). While these systems emulate the layered structure of cognition, they remain primarily statistical and lack transparent interpretability grounded in human-like thought processes.

### Hard versus easy problems of consciousness

 Shifts, and transitions in awareness. Although these processes are intricate, they are amenable to mathematical formalization. Our framework also tangentially touches the “hard problem”: how subjective experience (qualia) emerges from physical processes. While a challenge, we propose that recursively gated thresholds may provide structural prerequisites for conscious experience. Recursive formulations have appeared in both cognitive and cosmological models (Shin 2016; Shin and Kim 2017), including the use of recursive Heaviside functions to represent unresolved or parallel mental states (Shin 2014). Philosophers such as Schrödinger and Penrose (Schrödinger 1958; Penrose 1989) have raised fundamental questions about the mind—brain interface, while quantum cognitive theories (Busemeyer and Bruza 2012; Bruza et al. 2015) and neurodynamic synchronization frameworks (Varela et al. 2001) have emphasized systemic coherence.

The recursive sigmoid-based model we introduce is inspired by fuzzy logic (Zadeh 1965, 1975a; 1975b; 1975c), wherein graded transitions replace binary thresholds—a requirement for modeling the continuity of cognitive phenomena. This aligns with neurobiological evidence linking synaptic potentiation and memory formation to graded thresholds of activation (Kandel 2001).

## Supplementary discussion: philosophical background and Kantian time

Although the main body of our work focuses on a mathematical model for recursive cognition and memory, we briefly note here a conceptual inspiration from Immanuel Kant’s philosophy.

In the *Critique of Pure Reason*, Kant (1781) proposed that time is not an objective property of the external world, but a necessary *form of inner intuition* through which the mind organizes experiences. This view suggests that temporal experience is structured by the subject and unfolds as a recursive process of synthesis—constructing the flow of time from within, rather than receiving it externally.

While our model does not aim to directly implement Kantian metaphysics, this idea loosely resonates with our formulation of recursive memory thresholds ($\tau _i$) and cognitive layering. In our framework, each mental event is not simply placed at a point in time, but emerges recursively through internal thresholds—mirroring Kant’s suggestion that the temporal order of consciousness is generated by the subject’s structuring faculties.

We emphasize, however, that this analogy serves as a philosophical backdrop rather than a formal theoretical basis. Our model stands independently as a mathematical and cognitive framework, but the Kantian insight helps motivate why a recursively structured treatment of time might be psychologically and cognitively meaningful.

### Conceptual scope and contribution

To situate our model within broader theories of cognition, we draw connections to major frameworks in philosophy (Kant (1929); Chalmers (1995)), psychology (Freud (1900); Squire (2009)), neuroscience (Kandel (2001); Baddeley (2000)), and artificial intelligence (RNNs, attention-based systems). Rather than presenting the recursive Heaviside function as a universal solution, we show how its structure naturally reflects:

- Two distinct mechanisms of forgetting—decay of signal sharpness and overwriting by subsequent thresholds.
- Two modes of cognitive silence—recursive fade-out and active inhibition.

These examples suggest that the recursive Heaviside sequence function offers a mathematically tractable framework for modeling layered cognition, even as the interpretation of higher-order mixed derivatives remains an open question.

### Framing the methodological justification

While the recursive Heaviside framework was originally introduced to model threshold-based activations, its application to cognition is not a forced imposition of an existing tool. Rather, it emerges naturally from the observed structure of human memory, awareness, and recursive reflection.

We do not claim that this formulation universally explains all cognitive processes. Instead, it offers a mathematically precise approach for modeling consciousness as a layered, threshold-sensitive dynamic—one that responds to stress, attention, and recursive encoding.

In this sense, our framework is not a case of “a hammer looking for a nail,” but a carefully constructed lens designed for a class of phenomena that inherently exhibit recursive and graded activation patterns. The remainder of this paper explores how the recursive Heaviside model captures layered mental states, stress-modulated thresholds, and memory dynamics in a temporally distributed framework.

#### Contributions

 The central contribution of this paper lies in proposing a mathematically tractable model of recursive cognition based on smoothed Heaviside functions. This model captures both temporal unfolding and context-sensitive activation of memory layers, providing a formal structure for time-based awareness and recall dynamics.

#### Modeling constraints and temporal focus

 Our focus here is on time-based recursive cognition and human memory recall. Although this simplification omits spatial dynamics and nonlocal synchronization in the brain, it enables the formulation of a first-principles mathematical framework for recursive cognition. As Davies (1983) has noted, cognitive processes ultimately involve processes beyond time-only causality. Nonetheless, we treat our current model as a foundational structure that may be extended in future work to include distributed fields, spatial memory integration, and brain-wide synchrony.

#### Why no external time window is needed

 In conventional time-series models, memory or attention is often constrained using external time window functions—such as sliding kernels or decaying windows—to isolate recent past inputs from older ones. However, our recursive formulation inherently encodes temporal causality and irreversibility.

Each memory layer activates only after its corresponding threshold time $\tau _i$, as defined by the smoothed Heaviside function $H_{s_i}(-t + \tau _i)$. Since $H(-t + \tau _i) = 0$ for all $t < \tau _i$, no premature activation is mathematically possible. This means that recursive cognitive potentials are intrinsically gated in time: each layer can only respond after its causal trigger has occurred.

Thus, there is no need to introduce artificial windowing mechanisms. The recursive structure itself establishes a temporally bounded and hierarchically consistent memory activation scheme—one that mirrors the forward flow of subjective time.

This feature makes our model not only analytically clean but also cognitively plausible: it reflects the fact that humans cannot recall memories before they are formed, nor can they act upon events that have not yet happened. We always think in the present—even when we remember the past, the act of remembering takes place now.

#### Summary of contributions

 In this paper, we propose a Recursive Heaviside Memory Architecture in which cognitive layers are constructed through smooth threshold functions and nested derivatives. This framework:

- Captures how recursive awareness emerges and decays over time,
- Models memory thresholds and stress sensitivities,
- And identifies mathematical limits of reconstruction using time and parameter derivatives.

Through this approach, we aim to bridge cognitive modeling with rigorous recursive structures, offering an interpretable alternative to black-box neural models.

## Recursive structure of thought: self-tracking and recursive identity

### Recursive self-tracking and meta-awareness

One unique aspect of human cognition is its ability to observe itself. We not only perceive the external world but also become aware of our own thoughts, feelings, and memories. This capacity—often referred to as *self-tracking*—allows the mind to recursively monitor its internal states. It forms the basis for what we call a recursive identity, where the mind acts both as observer and observed. We model this structure using smooth step-like functions that activate when specific thresholds in time are crossed. Each layer of thought is defined recursively—depending on the activation of deeper layers. This nesting reflects how new thoughts or reflective awareness build upon earlier, often unconscious, mental states.

#### Why we use smooth functions

Before introducing the recursive equations, we address a key modeling decision. When using ideal (discontinuous) Heaviside functions, it is possible to write different recursive formulas that generate identical shapes but have different derivatives. This makes it difficult to analyze mental change or the dynamics of awareness. To avoid such ambiguity, we employ smooth sigmoid versions of the Heaviside function throughout this paper. This ensures that all recursive structures are differentiable and analytically consistent.

#### Recursive formulation of cognitive layers

Each recursive layer $h_i(t)$ is activated based on its own threshold $\tau _i$, sensitive (stress/sharpness/impressiveness) parameter $s_i$, and the output of the next deeper layer $h_{i+1}(t)$. The structure is defined as:

1 $$\begin{aligned} h_n(t)&= H_{s_n}\left( -t + \tau _n\right) , \nonumber \\ h_{n-1}(t)&= H_{s_{n-1}}\left( -t + \tau _{n-1} \cdot h_n(t)\right) , \nonumber \\ h_{n-2}(t)&= H_{s_{n-2}}\left( -t + \tau _{n-2} \cdot h_{n-1}(t)\right) , \nonumber \\&\quad \vdots \nonumber \\ h_1(t)&= H_{s_1}\left( -t + \tau _1 \cdot h_2(t)\right) , \nonumber \\ u_i(t)&= 1 - h_i(t), \quad i = 1,\dots ,n \end{aligned}$$

Here, $u_1(t)$ represents present awareness, while deeper layers $u_2(t), u_3(t), \dots , u_n(t)$ encode unconscious memories, emotions, or prior perceptions. The output of each layer depends recursively on the layers beneath it, capturing the accumulation and modulation of mental state.

#### Time-sensitive recursive dynamics

We define the recursive function compactly as:

$$ u_n(t; \tau _1, \tau _2, \ldots , \tau _n) $$

indicating that the mental potential at time $t$ depends on a history of threshold-crossing events. The dynamic behavior of this structure is governed by a combination of temporal and threshold sensitivities. By inspection,we propose the following differential equation to model these interactions:

$$ (-1)^k \frac{\partial u_k}{\partial t} + \sum _{i=k}^{n} \frac{\partial u_k}{\partial \tau _i} = 0, \quad \text {for } k = 1, \dots , n $$

This expression captures the recursive influence of threshold-based memory layers on temporal change. The alternating sign structure reflects the hierarchical dynamics of awareness modulation, where some cognitive layers reinforce signal flow while others suppress or mask it.

### Relation to the multidimensional advection equation

 This recursive structure suggests a deeper connection to a generalized conservation principle. In the limit where each Heaviside function becomes perfectly sharp, the mental potential simplifies to an exact recursive form:

$$ u(t) = 1 - H(-t + \tau _1 H(-t + \tau _2 H(-t + \tau _3 \cdots ))), $$

and in this case, it satisfies a formal multidimensional advection equation:

$$ (-1)^k \frac{\partial u_k}{\partial t} + \sum _{i=k}^{n} \frac{\partial u_k}{\partial \tau _i} = 0, \quad \text {for } k = 1, \dots , n $$

where $t$ is the time axis and each $\tau _i$ is a recursive threshold coordinate. This equation represents a balanced information flow in the cognitive state space, as if each threshold modulates memory dynamics in a conservative manner. However, this identity holds strictly only in the idealized limit of discontinuous transitions. When we instead consider recursive sigmoid sequences, replacing Heaviside steps with:

$$ H_{s_i}(x) = \frac{1}{1 + e^{-2x/s_i}} $$

the function $u(t)$ becomes continuous and differentiable—but the sharp thresholding that guarantees the advection structure is lost. Finite sensitivity (stress/sharpness/impressiveness) parameters $s_i$ introduce temporal smoothing and blurring of threshold boundaries, resulting in leakage between layers and violating the conservation-like behavior.

Thus, the advection-like differential identity is exact only in the singular case of perfect step transitions. In more realistic sigmoid-based models, the same structural equation no longer holds, revealing fundamental limitations in reconstructing or conserving recursive memory flow.

#### Recursive layers as parallel universes

 While many cognitive models treat memory layers as interacting components of a unified mind, our recursive formulation allows for a stronger interpretation: Each layer $u_k(t)$ evolves based on its own threshold $\tau _k$ and recursively gated prior layers, but it does not retroactively affect them.

This asymmetric causality, combined with the non-overlapping activation domains enforced by Heaviside functions, suggests a structure in which each memory layer acts as a temporally bounded, quasi-independent entity. Thus, these recursive strata can be viewed as constituting parallel mental universes—not merely metaphorical, but mathematically defined and causally disjoint within the recursive flow of time.

Unlike classical parallel universe theories in physics that posit branching at quantum events, our structure yields *recursive parallelity*: new branches are not splits but nested delays, each waiting to unfold upon crossing its own threshold.

This view aligns with interpretations of cognitive recursion as layered realities and extends to metaphysical implications about memory, identity, and layered existence.

#### Emergence of meta-cognition

In special cases, a threshold $\tau _i$ may become dependent—through recursion—on its own effect. This feedback marks the emergence of true meta-awareness: the capacity to *be aware of being aware*. Such moments occur when the system references not only external events but also internal recursive states.

This recursive feedback loop defines a self-referential identity: a mental architecture capable of tracking, modifying, and reflecting upon itself. Moments of recalling a memory, noticing that we are doing so, or questioning why we feel a certain way all reflect the emergence of recursive cognition and meta-memory.

#### Summary

In summary, this recursive, soft-threshold-based model captures how human minds build and track layered thoughts over time. The use of smooth sigmoid functions ensures analytic clarity, while the recursive dependency structure offers a compact yet powerful way to describe self-aware mental dynamics. In the following sections, we expand this model to simulate attention, forgetting, and cognitive branching.

#### Self-tracking structure and temporal windowing

 To model how thoughts and memories emerge in a structured sequence, we use a recursive architecture built from smooth Heaviside functions. The cognitive potential at each layer is defined as:

2 $$\begin{aligned} u_n(t)&= 1 - H_{s_n}(-t + \tau _n) \nonumber \\ u_{n-1}(t)&= 1 - H_{s_{n-1}}\left( -t + \tau _{n-1} \cdot H_{s_n}(-t + \tau _n)\right) \nonumber \\&\vdots \nonumber \\ u_1(t)&= 1 - H_{s_1}\left( -t + \tau _1 \cdot H_{s_2}\left( -t + \tau _2 \cdots \right) \right) \end{aligned}$$

Each layer activates only after the deeper one has already triggered—similar to how certain memories surface only after recalling related prior experiences. This structure creates a form of *temporal windowing*, where each threshold reveals when and how it contributes, depending on whether previous conditions are met.

This layered gating reflects how our minds filter and structure information across time, supporting shifts in awareness, sequential memory recall, and higher-order thought.

## Defining the recursive Heaviside function

At the heart of our model lies a recursive structure composed of nested Heaviside functions. This formulation captures how mental potential grows through successive cognitive thresholds, each of which must be crossed in a specific order to activate higher-level mental states.

### Smoothing for analytic clarity

While the ideal Heaviside step function is conceptually clear, its discontinuity makes it unsuitable for differential analysis. Moreover, distinct recursive structures can produce identical output curves while yielding different derivatives—posing challenges for interpretability. To address this, we adopt smoothed sigmoid versions of Heaviside functions throughout this paper.

Each smoothed function is defined as:

3 $$\begin{aligned} \sigma _s(x) = \frac{1}{1 + \exp \left( -\frac{2x}{s}\right) } \end{aligned}$$

Here, the sensitivity (stress/sharpness/impressiveness) parameter $s$ controls the sharpness of the transition: small $s$ yields a steeper (more Heaviside-like) step, while large $s$ leads to a smoother and more gradual slope. We interpret $s_i$ as the sensitivity (stress/sharpness/impressiveness) of the $i$-th layer—smaller values reflect abrupt responses (e.g., trauma), while large values correspond to slower, longer-lasting cognitive transitions.

### (a) Explicit nested form

The recursive function $u(t)$ encodes cognition as a series of nested threshold activations. Each tier corresponds to a cognitive layer that becomes active when its associated condition is met. Rather than enumerating each layer explicitly, we capture the recursive hierarchy through the compact functional form and accompanying diagrams.

Using sigmoid functions, the recursive cognitive potential can be written in fully expanded nested form. For example, for a 4-layer structure:

4 $$\begin{aligned} u_4(t)&= 1 - \frac{1}{1 + \exp \left( -\dfrac{2}{s_4}(-t + \tau _4) \right) } \nonumber \\ u_3(t)&= 1 - \frac{1}{1 + \exp \left( -\frac{2}{s_3} \left( -t + \tau _3 \cdot \frac{1}{1 + \exp \left( -\frac{2}{s_4}(-t + \tau _4) \right) } \right) \right) } \nonumber \\ u_2(t)&= 1 - \frac{1}{1 + \exp \left( -\frac{2}{s_2} \left( -t + \tau _2 \cdot \frac{1}{1 + \exp \left( -\frac{2}{s_3} \left( -t + \tau _3 \cdot \cdots \right) \right) } \right) \right) } \nonumber \\ u_1(t)&= 1 - \frac{1}{1 + \exp \left( -\frac{2}{s_1} \left( -t + \tau _1 \cdot \frac{1}{1 + \exp \left( -\frac{2}{s_2} \left( -t + \tau _2 \cdots \right) \right) } \right) \right) } \end{aligned}$$

This formulation emphasizes the depth of recursion: each layer’s activation depends on all prior layers being at or near their respective thresholds. While analytically valid, this form becomes impractical for deeper models and is better interpreted conceptually than computationally.

The general form of the recursive cognitive potential, which exhibits a continued fraction structure with infinite nested thresholds, is provided in Appendix A. Here, we show only the four-layer case for illustrative purposes.

### (b) Recursive function definition

Alternatively, the same structure can be expressed recursively through a sequence of nested sigmoid functions:

5 $$ \begin{aligned} h_{n} (t) = & \frac{1}{{1 + \exp \left( { - \frac{2}{{s_{n} }}\left( { - t + \tau _{n} } \right)} \right)}}, \\ h_{i} (t) = & \frac{1}{{1 + \exp \left( { - \frac{2}{{s_{i} }}\left( { - t + \tau _{i} \cdot h_{{i + 1}} (t)} \right)} \right)}},i = n - 1, \ldots ,1, \\ u_{i} (t) = & 1 - h_{i} (t),\quad i = 1, \ldots ,n. \\ \end{aligned} $$

This formulation highlights the recursive nature of cognition, where each layer is modulated not only by its own temporal threshold $\tau _i$ and sensitivity (stress/sharpness/impressiveness) parameter $s_i$, but also by the activation level of the next deeper layer. The structure models how higher-order cognitive states emerge through recursive integration of prior mental layers, allowing each layer to serve as both an output and a gate for subsequent processing.

We interpret $h_i(t)$ as the activation state of the $i$-th cognitive layer, which is recursively shaped by the deeper layer $h_{i+1}(t)$. The variable $u_i(t) = 1 - h_i(t)$ then represents the residual potential—mental content that remains unactivated until triggered. The total number of recursive layers, $n$, can be interpreted as a proxy for the complexity or depth of recursive processing, offering a conceptual correlate of what may be termed the “recursive depth of consciousness.” This recursive model thus provides a principled and interpretable framework for simulating time-dependent processes such as attention, self-awareness, and context-sensitive memory encoding.

### Memory projection onto the time axis

In the Recursive Heaviside Memory Model (RHMM), memory is not encoded as a discrete event localized at a single time point. Instead, it emerges as a *graded cognitive potential* distributed across the temporal domain. Each smoothed sigmoid function $H_{s_i}(x)$ defines a continuous activation over a finite time window, with its instantaneous value contributing to the evolving composite memory signal.

This approach contrasts with sharp Heaviside-based models, in which mental events are modeled as abrupt, isolated transitions. In RHMM, by contrast, memory traces manifest as *segments* projected along the time axis—each shaped by the slope and breadth of its corresponding sigmoid. The continuity of these projections allows mental states to span durations and vary in intensity, rather than being confined to momentary spikes.

The vertical profile of a sigmoid’s projection can be interpreted as indicating the **strength and duration** of a mental imprint. Sharper sigmoids (small $s_i$) correspond to brief, high-intensity cognitive episodes—such as trauma, sudden awareness, or abrupt attention shifts. In contrast, smoother sigmoids (large $s_i$) reflect prolonged, low-intensity states, such as background thoughts or gradually forming emotions.

This framework offers a novel visualization of recursive cognition: as overlapping, nested fields of mental activity evolving continuously through time. Each layer’s activation depends not only on its temporal threshold $\tau _i$ but also on its sensitivity (stress/sharpness/impressiveness) parameter $s_i$, which modulates the degree of emotional charge, stress intensity, or attentional salience.

Ultimately, RHMM conceptualizes memory as a dynamic, stress-sensitive waveform—layered recursively and projected across time. This representation forms the foundation for subsequent analyses of attention, recall, and temporal awareness. Ultimately, RHMM portrays memory not as a static point but as a dynamic waveform—layered, continuous, and stress-sensitive—projected across recursive time.^1^

## Recursive structure of thought: self-tracking and recursive identity

### Layered cognitive potentials: high-stress example

To illustrate the recursive structure of cognition under significant stress conditions, we simulate an 8-layer recursive potential using moderate-to-large sharpness parameters $s_i$, modeling the accumulation of unresolved emotional experiences over the course of a life. Each threshold $\tau _i$ corresponds to a cognitively significant life event distributed across an 80-year timespan:

$$\begin{aligned} & \tau _1 = 25.25,\quad \tau _2 = 37.5,\quad \tau _3 = 43.75,\quad \tau _4 = 51.25,\\ & \quad \tau _5 = 62.5,\quad \tau _6 = 67.5,\quad \tau _7 = 70.0,\quad \tau _8 = 80.0 \quad \text {(years)} \end{aligned}$$

The sensitivity (stress/sharpness/impressiveness) values $S_i$ are chosen as:

$$ S_i = [11.1,\ 53.3,\ 33.2,\ 22.5,\ 4.1,\ 14.1,\ 24.1,\ 8.0] $$

Each layer represents recursive cognitive activation triggered by emotional thresholds. The sigmoid function $H_{s_i}(x)$ controls the sharpness and duration of memory formation.

**Fig. 2:**
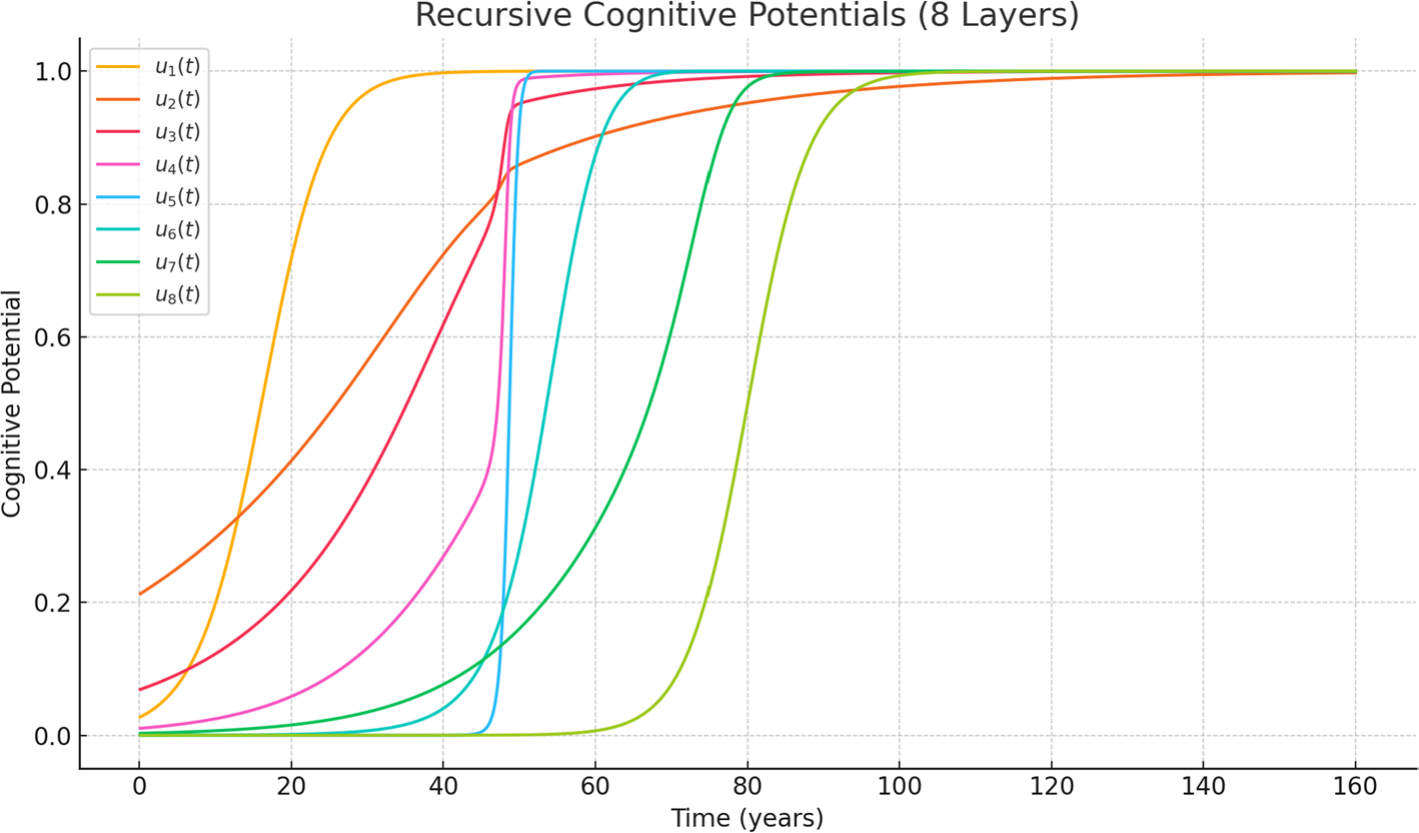
Recursive potential with large $s_i$ values (high-stress/overload regime). Each sigmoid layer transitions gradually after its threshold $\tau _i$, resulting in a diffused profile. This corresponds to persistent memory encoding under emotionally overloaded conditions

To simplify the presentation and focus on a single case, we have removed the low-stress example and retained only the high-stress configuration shown in Fig. 2.

As illustrated, large $s_i$ values cause the sigmoid transitions to become broad and overlapping. This produces long-lasting mental potentials, modeling the recursive influence of unresolved emotions or persistent attentional traces. Such a recursive profile may help conceptualize phenomena such as trauma echo, chronic ruminations, or the lasting effects of formative events.

### Stress sharpness and memory persistence

The sharpness parameter $s_i$ modulates the width and intensity of each memory layer:

- *Large* $s_i$: flat sigmoid transitions $\rightarrow$ slow onset and long decay $\rightarrow$ persistent cognitive potential.
- *Small* $s_i$: sharp sigmoid transitions $\rightarrow$ fast onset and self-cancellation $\rightarrow$ fleeting awareness.

sharp sigmoid transitions $\rightarrow$ fast onset and self-cancellation $\rightarrow$ fleeting awareness.

In this section, we focus on the persistent case (large $s_i$), which captures the recursive buildup of unresolved or repetitive internal activations—a mathematical analog of chronic memory traces or psychological entanglement.

The inverse relationship between $s_i$ and cognitive resolution emerges naturally from the recursive structure: the broader the sigmoid, the more distributed its influence, and the less temporally specific the awareness. This provides insight into how stress can modulate not only emotional intensity but the structural layering of cognition itself.

#### Philosophical reflection

 In our recursive memory architecture, the presence of stress-sensitive thresholds implies that no cognitive trace is ever truly neutral. Each mental event, by passing a threshold $\tau _i$ under a nonzero stress level $s_i$, forms a recursive layer that persists—often beyond conscious awareness. This leads us to a sobering view: To have a body is to experience stress. To experience stress is to embed memory recursively. And to embed memory is to inherit one’s own past, even beyond a single lifetime. In this sense, as one might say in poetic terms:

To live with flesh is to decay from it. To be born human is to carry layered burdens—thresholds never asked for, but always remembered.

## Proposed methodology

A key element of our approach is the use of the Recursive Heaviside Sequence Function, capturing threshold-based activation in cognition:

6 $$\begin{aligned} u(t) = 1 - H_{s_1}\left( -t + \tau _1 H_{s_2}\left( -t + \tau _2 H_{s_3}\left( -t + \tau _3 \cdots \right) \right) \right) \end{aligned}$$

In this formulation:

- $H_{s_i}$ is a smoothed Heaviside function that models the transition at cognitive layer *i*. It is parameterized by sensitivity (stress/sharpness/impressiveness) level $s_i$, which controls how sharp or gradual the activation is. The function is approximated using a sigmoid:
  $$ H_s(x) = \frac{1}{1 + \exp (-2x/s)} $$
- $\tau _i$ represents the cognitive threshold time at layer *i*, marking when awareness or memory encoding occurs.

This nested structure forms a conditional activation hierarchy: each layer activates only if all inner layers have already crossed their respective thresholds. This recursive dependency models key cognitive mechanisms such as introspective awareness, conditional memory formation, and temporal gating. We refer to this entire structure as the Recursive Heaviside Memory Model (RHMM).

The RHMM framework supports simulation of several core cognitive functions:

- *Recursive memory accumulation*: Memory is stored progressively across recursive layers.
- *Attention-gated activation*: Higher layers engage only when certain thresholds are met in time.
- *Self-monitoring through recursion*: Each layer adjusts based on the state of previous inner layers.

### Why recursive Heaviside?

The Recursive Heaviside formulation is not chosen arbitrarily. It models how mental events accumulate conditionally and nonlinearly in time. Traditional memory models often assume either linear accumulation or isolated encoding, but our formulation captures:

- *Conditional cognition*: Later awareness depends on prior thresholds being crossed.
- *Stress-sensitive transitions*: Sharper memories leave steeper traces in the cognitive field.
- *Recursive accumulation*: Cognitive layers encode layers of reflection, akin to introspection.

This recursive, gated architecture makes the RHMM especially suited to modeling phenomena such as trauma encoding, sudden awareness, and layered memory states.

### Memory storage mechanism

Unlike traditional systems that rely on explicit “save” operations or fixed memory addresses, the RHMM encodes memory as an emergent property of recursive modulation. When a cognitive event occurs at time *t*, it is embedded into the recursive structure by:

- Adjusting the recursive thresholds $\tau _i$, which represent moments of attention or awareness.
- Modulating the sensitivity (stress/sharpness/impressiveness) parameters $s_i$, which capture the emotional or perceptual intensity of the experience.

This mechanism produces memory traces that:

- Are distributed across recursive layers, rather than stored at discrete locations.
- Can be dynamically reinforced or gradually weakened, depending on the interplay between recursive reactivation and new inputs.

The recursive hierarchy naturally enforces a temporal stratification:

- Older memories are embedded deeper and require greater computational effort (e.g., higher-order derivatives) to recall, but tend to be more stable.
- Recent experiences reside in outer layers, making them more accessible but also more susceptible to modification or interference.

In this way, the Recursive Heaviside Memory Model (RHMM) provides a biologically inspired, temporally layered, and emotionally modulated memory architecture that contrasts sharply with conventional address-based systems.

Table 1 presents a comparison between conventional memory systems and the Recursive Heaviside Memory Model (RHMM), emphasizing RHMM’s temporal recursion and emotional layering as core cognitive features.

**Table 1:** Comparison of conventional memory architectures and the recursive heaviside memory model

Aspect	Conventional Memory (e.g., RAM, AI models)	Recursive Heaviside Memory Model
Storage structure	Address-based; fixed memory locations	Threshold-based recursive layers; evolves with time
Access mechanism	Direct retrieval via addresses or indices	Recall via recursive differentiation w.r.t. time, threshold, or stress parameter
Clonability	Exact duplication possible	Non-clonable due to recursive dependency
Modifiability	Direct overwriting at known locations	Modulated via recursive interaction and threshold shifts
Persistence	Static; remains unless overwritten	Dynamic; reinforced or faded through recursive reactivation
Encoding parameters	Bits, vectors, or weights	Thresholds $\tau _i$, sensitivity (stress/sharpness/impressiveness) parameters $s_i$
Recall behavior	Deterministic and isolated	Context-sensitive; shaped by recursive layering
Time sensitivity	Typically time-agnostic	Strongly time-dependent; deeper layers encode older memories
Data representation	Discrete memory blocks	Distributed dynamic potentials over recursive structure

## Type 1: time derivative structure satisfying the non-conservative advection equation

For the recursive cognitive potential $u_k(t)$ at layer $k$, the time derivative term must be defined with a layer-specific sign $(-1)^{k+1}$ to match the recursive advection structure:

7 $$\begin{aligned} (-1)^{k} \frac{\partial u_k}{\partial t}&= (-1)^{k+1} \cdot \left( -\delta _{s_k}(A_k) \cdot \frac{\partial A_k}{\partial t} \right) \end{aligned}$$

8 $$\begin{aligned}&= \delta _{s_k}(A_k) \cdot (-1)^k \cdot \frac{\partial A_k}{\partial t} \end{aligned}$$

The recursive expression for the rate of change of $A_k$ is:

$$ \frac{\partial A_k}{\partial t} = -1 - \tau _k \cdot \delta _{s_{k+1}}(A_{k+1}) \cdot \frac{\partial A_{k+1}}{\partial t} $$

with the deepest layer satisfying:

$$ \frac{\partial A_n}{\partial t} = -1 $$

As a concrete example, the outermost layer $u_1(t)$ is defined as:

$$\begin{aligned} & u_1(t) = 1 - H_{s_1}(A_1), \quad A_k = -t + \tau _k H_{s_{k+1}}(A_{k+1}), \\ & \quad A_n = -t + \tau _n \end{aligned}$$

Its time derivative follows the recursive chain:

9 $$\begin{aligned} \frac{\partial u_1}{\partial t}&= -\delta _{s_1}(A_1) \cdot \frac{\partial A_1}{\partial t} \end{aligned}$$

10 $$\begin{aligned} \frac{\partial A_k}{\partial t}&= 1 + \tau _k \cdot \delta _{s_{k+1}}(A_{k+1}) \cdot \frac{\partial A_{k+1}}{\partial t}, \quad k = 1, \dots , n-1 \end{aligned}$$

11 $$\begin{aligned} \frac{\partial A_n}{\partial t}&= -1 \end{aligned}$$

Therefore, the full derivative becomes:

$$ \begin{aligned} \frac{{\partial u_{1} }}{{\partial t}} = & - \delta _{{s_{1} }} (A_{1} ) \cdot \left( { - 1 - \tau _{1} \delta _{{s_{2} }} (A_{2} ) \cdot } \right. \\ & \quad \left. {\left( { - 1 - \tau _{2} \delta _{{s_{3} }} (A_{3} ) \cdots \left( { - 1 - \tau _{{n - 1}} \delta _{{s_{n} }} (A_{n} )} \right) \cdots } \right)} \right) \\ \end{aligned} $$

The term $(-1)^{k+1} \frac{\partial u_k}{\partial t}$ restores the correct sign for the non-conservative advection equation structure.

**Fig. 3:**
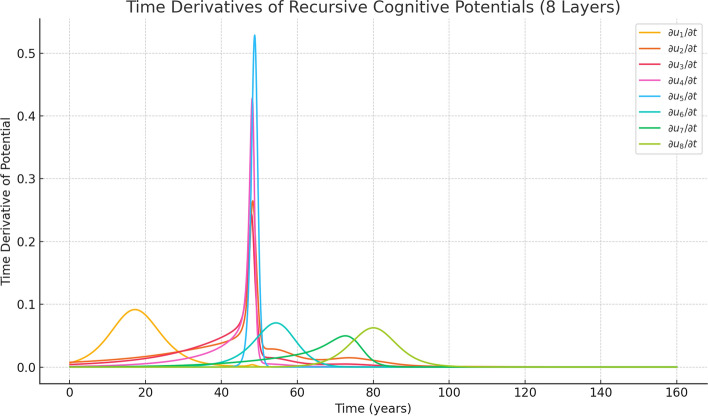
Time derivative of recursive cognitive potentials $$u_1(t)$$ through $$u_8(t)$$. Highersensitivity (stress/sharpness/impressiveness) values $$s_i $$ yield smoother, more extended transitions (e.g., $$s_5 = 4.1 $$), whereas lower values produce sharper, short-lived transitions. These transitions represent a "fuzzy" form of mental potential, reflecting distributed awareness across time rather than instantaneous jumps. The x-axis denotes time (years), and the y-axis represents mental potential

Figure 3 shows the time derivatives of the recursive cognitive potential functions for the soft case (high sensitivity (stress/sharpness/impressiveness), large $$_si $$). Each layer exhibits broader and longer-lasting activation, indicating persistent but diffuse awareness across time.

$$ \frac{\partial u_1}{\partial t}, \quad \frac{\partial u_2}{\partial t}, \quad \ldots , \quad \frac{\partial u_8}{\partial t} $$

- Deeper layers display sharper, delayed spikes due to their hierarchical depth.
- These spikes correspond to transient awareness events—brief yet intense cognitive activations.

## Type 2: threshold derivative: attention-driven recall

In this model, retrieval is also driven by the partial derivative with respect to cognitive thresholds $\tau _i$:

$$ \frac{\partial u_j}{\partial \tau _i}, \quad j = 1, \ldots , i-1 $$

We propose a recursive advection equation that incorporates threshold derivatives:

$$ (-1)^{k} \frac{\partial u_k}{\partial t} + \sum _{i=k}^{n} \frac{\partial u_k}{\partial \tau _i} = 0, \quad \text {for } k = 1, \ldots , n $$

Here, $H(-t + \tau _i)$ serves as a gating function—activating retrieval only after the corresponding threshold time is reached. This enforces **causal retrieval**: cognition unfolds forward, and prior memories become accessible only after encoding.

The term $\frac{\partial u_j}{\partial \tau _i}$ reveals how attentional encoding at layer $i$ modulates mental potential at layer $j$, exhibiting:

- *Temporal locality*: strongest near $t = \tau _i$,
- *Vertical attenuation*: influence weakens as the signal moves up,
- *Causal asymmetry*: deeper layers ($j > i$) remain unaffected by later thresholds.

We define:

$$ \begin{gathered} u_{j} (t) = 1 - H_{{s_{j} }} (A_{j} ), \hfill \\ {\text{where }}A_{j} = - t + \tau _{j} H_{{s_{{j + 1}} }} (A_{{j + 1}} ), \hfill \\ j = 1, \ldots ,n \hfill \\ \end{gathered} $$

This structure models how attentional thresholds sculpt recursive recall in a forward-propagating and temporally gated manner.

### Threshold derivatives $\partial u_j / \partial \tau _i$

To quantify how each recursive layer responds to an attentional change at threshold $\tau _i$, we compute its partial derivative:

#### Convention

For notational and computational consistency, we define the initial threshold as ${\tau}_0 =1$. This ensures the recursive structure remains well-defined and aligns with numerical implementations where indexing begins at zero. It also provides a normalized reference for temporal encoding.

12 $$\begin{aligned} \frac{\partial u_j}{\partial \tau _i}=0 \qquad\qquad \qquad\qquad \qquad\qquad\text {if } j> i, \end{aligned}$$

13 $$\begin{aligned} \frac{\partial u_j}{\partial \tau _i}&= \left( \prod _{k=j}^{i} \tau _{k-1} \cdot \delta _{s_k}(A_k) \right) \cdot H_{s_{i+1}}(A_{i+1}), \quad \text {if } j \le i < n, \end{aligned}$$

14 $$\begin{aligned} \frac{\partial u_j}{\partial \tau _i}&= \left( \prod _{k=j}^{n} \tau _{k-1} \cdot \delta _{s_k}(A_k) \right), \qquad   \quad \text {if } j \le i = n. \end{aligned}$$

These expressions highlight the recursive, threshold-sensitive nature of cognitive structure:

- For $j > i$: the derivative passes through intermediate delta gates and is filtered by the next-layer Heaviside.
- For $j = i$: the Heaviside term $H_{s_{i+1}}(A_{i+1})$ is typically close to 1 and may be omitted in approximation.
- For $j > i$: the response is zero—deeper layers are not influenced retroactively due to causal gating.

This confirms that attentional recall travels upward only—lower (deeper) memory layers remain stable unless explicitly triggered.

**Fig. 4:**
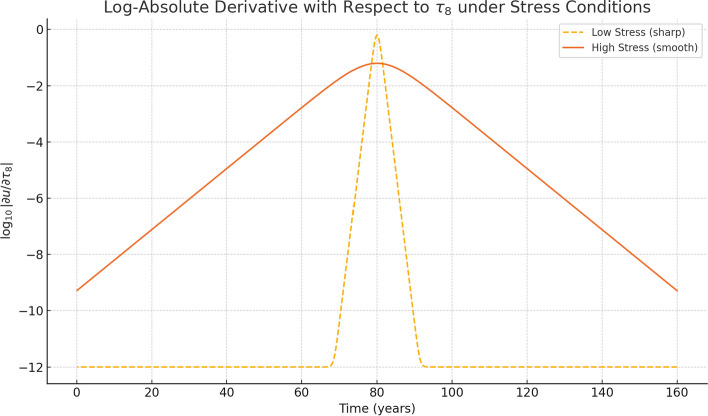
Logarithmic plot of absolute values of the threshold derivative $\left| \partial u / \partial \tau _8 \right|$ under soft and hard conditions. High-stress conditions produce narrow peaks; soft stress results in longer-duration activations. The logarithmic scale reveals fine-grained modulation across layers and conditions

Figure 4 presents the logarithm of the absolute threshold derivative under both soft and hard stress conditions.

- In the hard condition (high stress or sharply gated attention), the $\tau _8$, indicating strong but short-lived memory activation.
- In soft conditions, activation is weaker but more persistent, reflecting prolonged, mild retention.

We apply the logarithmic scale to visualize both cases on a comparable scale. Without it, soft activation would appear negligible. The transformation clarifies that high-stress attention causes narrow spikes, while soft stress yields prolonged, mild activation.

## Type 3: stress derivative—sensitivity to cognitive pressure

While the time derivative $\frac{\partial u}{\partial t}$ and threshold derivative $\frac{\partial u}{\partial \tau _i}$ describe temporal awareness and attentional recall, the stress derivative $\frac{\partial u}{\partial s_i}$ reflects how sensitive the cognitive structure is to variations in pressure or intensity at each layer.

The sharpness of each recursive layer is governed by the sensitivity (stress/sharpness/impressiveness) parameter $$s_i $$, which determines how abruptly the mental potential transitions at threshold $$ \tau _i $$. The smoothed Heaviside function is defined as:

$$ H_{s_i}(x) = \frac{1}{1 + \exp \left( -\frac{2x}{s_i}\right) } $$

Differentiating the overall cognitive potential $u(t)$ with respect to $s_{i}$ quantifies how changes in mental stress levels reshape the memory curve:

- Small $s_{i}$: corresponds to low stress or traumatic encoding—leading to sharp, spike-like memory activation.
- Large $s_{i}$: reflects strong or distributed stress—yielding smoother, broader cognitive responses.
- $\partial u / \partial s_i$ tracks how changes in sharpness affect the recursive engagement of layers.

Mathematically, the derivative chain follows a structure similar to the threshold derivative:

$$\frac{\partial u}{\partial s_3} = \frac{\partial H_{s_1}(A_1)}{\partial A_1} \cdot \tau _1 \cdot \frac{\partial H_{s_2}(A_2)}{\partial A_2} \cdot \tau _2 \cdot \frac{\partial H_{s_3}(A_3)}{\partial s_3} $$

This means that stress derivatives are not fundamentally new quantities—they mirror the threshold derivatives in structure and effect. Thus, the qualitative behavior of $\frac{\partial u}{\partial s_i}$ is already captured by our analysis of $\frac{\partial u}{\partial \tau _i}$.        

Therefore, we do not present separate plots for $\frac{\partial u}{\partial s_i}$, as they visually and structurally replicate those of $\frac{\partial u}{\partial \tau _i}$. This redundancy emphasizes a deeper insight: in our recursive model, changes in *when* memory is encoded ($\tau _i$) and changes in *how sharply* it is encoded ($s_{i}$) are mathematically entangled and modulate the same recursive pathways.

### Irreversibility in exact recursive heaviside memory functions

In the limiting case where $$s_i \rightarrow 0 $$, the smoothed sigmoid function $$H_{s_i}(x) $$ converges to an exact Heaviside step function. This transforms the recursive memory architecture into a cascade of instantaneous, discontinuous switches—each layer encoding an irreversible binary decision.

Unlike smooth models that support gradient flow and allow for partial reversal or modulation, the exact Heaviside formulation eliminates any possibility of interpolation or reactivation. Once a cognitive threshold $\tau _i$ is crossed, the corresponding mental transition becomes permanently encoded.

In the limit of infinite layers ($n \rightarrow \infty$), the recursive structure approximates a deep binary tree of mental events, each embedded irreversibly in the timeline of cognition. This formulation mathematically explains why certain high-stress or traumatic events become deeply etched and resistant to modification. Such events behave as non-reversible attractors in the cognitive system, limiting the brain’s ability to reinterpret or weaken the associated memory trace over time.

#### Time derivative

In the exact recursive Heaviside formulation, the time derivative simplifies dramatically. Regardless of the recursion depth $n$, the nested structure collapses except at the outermost discontinuity. Specifically, we obtain:

$$ (-1)^n \frac{du_n}{dt} = \delta (-t + \tau _1). $$

This expression shows that the time derivative loses all information about deeper cognitive thresholds $\tau _2, \tau _3, \ldots$. Only the first activation point survives as a delta function, making the internal recursive structure invisible to time differentiation.

#### Derivatives with respect to thresholds

The recursive advection equation:

$$ (-1)^{k} \frac{\partial u_k}{\partial t} + \sum _{i=k}^{n} \frac{\partial u_k}{\partial \tau _i} = 0, \quad \text {for } k = 1, \dots , n, $$

provides the governing relation between time and threshold derivatives.

*Convention: Let* $$ \tau _0 = 1 $$.

The explicit form of the partial derivative with respect to a threshold $\tau _i$ is given by:

15 $$\begin{aligned} \frac{\partial u_j}{\partial \tau _i}&= \left( \prod _{k=j}^{i} \tau _{k-1} \cdot \delta _{S_k}(A_k) \right) \cdot H_{S_{i+1}}(A_{i+1}), \quad \text {if } j \le i < n. \end{aligned}$$

This expression contains nested products of delta functions evaluated at recursive arguments. However, such products are not defined in classical distribution theory (Colombeau 1984). Specifically, objects like $\delta (x)\delta (y)$ or $\delta (x)^k$ are not members of $\mathcal {D}'(\mathbb {R})$, rendering the expression formally ill-defined.

#### Interpretational limitation

Although the exact recursive Heaviside memory function is well-defined in closed form, its derivatives:

- Fail to retain the hierarchical structure encoded by internal thresholds $\tau _2, \tau _3, \ldots$,
- Degenerate into singularities localized at the outermost layer (e.g., $\tau _1$),
- Are non-invertible due to the undefined nature of delta products in standard distribution theory.

Some formal approaches, such as Colombeau algebras (Colombeau 1984), provide regularized frameworks for dealing with such expressions, but they do not preserve the original cognitive interpretability.

*Thus, the exact recursive Heaviside formulation encodes cognition in a fundamentally irreversible way. Its derivatives lose internal structure, degenerate to edge singularities, and render the memory architecture non-invertible.*

##### Recursive sigmoid case: smooth but still non-invertible

To overcome the mathematical limitations of the exact Heaviside case—where delta function products are ill-defined—we now consider a smoothed recursive formulation using sigmoidal approximations. Specifically, we define the memory function as:

$$ \begin{aligned} u_{n} (t) = & 1 - H_{{s_{1} }} \left( { - t + \tau _{1} H_{{s_{2} }} \left( { - t + \tau _{2} H_{{s_{3} }} \left( { - t} \right.} \right.} \right. \\ & \quad \left. {\left. {\left. { + \tau _{3} \cdots H_{{s_{n} }} \left( { - t + \tau _{n} } \right) \cdots } \right)} \right)} \right), \\ \end{aligned} $$

where $H_{s_i}(x) = \frac{1}{1 + e^{-2x / s_i}}$ denotes a sigmoid function with sensitivity (stress/sharpness/impressiveness) parameter $s_i > 0$. As $s_i \rightarrow 0$, the function approaches a step; large $s_i$ yields a gradual transition.

This smooth formulation resolves the issue of undefined products and allows all derivatives,

$$ \frac{\partial u_n}{\partial t}, \quad \frac{\partial u_n}{\partial \tau _i}, \quad \frac{\partial u_n}{\partial s_i}, $$

to be rigorously defined. However, despite this analytical tractability, the recursive structure introduces deep coupling between layers. Each derivative depends on the nested composition of previous sigmoid functions and their parameters, making the internal configuration opaque.

Even with complete differentiability, the recursive sigmoid memory remains fundamentally non-invertible: small perturbations in parameters $\{ \tau _i \}, \{ s_i \}$, or lack of knowledge about any internal layer, leads to large uncertainty in reconstructing the original potential. Thus, the function’s outward appearance (its derivative) fails to uniquely determine its internal structure.

**Special Case:** $n = 1$ As a notable exception, when the recursion depth is one, the potential reduces to a simple sigmoid:

$$ u_1(t) = 1 - H_{s_1}(-t + \tau _1), $$

whose derivative is unimodal:

$$ \frac{\partial u_1}{\partial t} = \frac{2}{s_1} \cdot \frac{e^{-2(-t + \tau _1)/s_1}}{\left( 1 + e^{-2(-t + \tau _1)/s_1}\right) ^2}. $$

This case permits closed-form recovery of both $\tau _1$ and $s_1$, as they are encoded in the location and shape of the peak. However, for $n \ge 2$, such recovery becomes infeasible.

**Conclusion** The recursive sigmoid model provides mathematical well-posedness, but not invertibility. Both exact and smoothed recursive memory functions exhibit the same fundamental limit: *Derivatives do not suffice to reconstruct the memory function without full knowledge of internal parameters.*

## Two ways the mind goes silent: from forgetting to trauma

Memory can fail to form for two opposite reasons: (1) when a situation feels unimportant, or (2) when the experience is overwhelmingly stressful. Though these may seem unrelated, our recursive sigmoid model explains both phenomena using the same mathematical structure. In both cases, the partial derivative

$$ \frac{\partial u}{\partial \tau _i}(t) $$

which typically indicates memory activation at a threshold $\tau _i$, becomes nearly zero. This signals that no memory is encoded.

### Case 1: Low stress—forgetting simple things

When the sensitivity (stress/sharpness/impressiveness) parameter $s_i \ll 1$, each sigmoid layer becomes steep and narrow, effectively approximating a Heaviside step function.

**Recursive Structure (5-Layer Example):**

$$ u(t) = 1 - H_{s_1}\left( A_1\right) $$

where

$$\begin{aligned} A_1&= -t + \tau _1 H_{s_2}(A_2) \\ A_2&= -t + \tau _2 H_{s_3}(A_3) \\ A_3&= -t + \tau _3 H_{s_4}(A_4) \\ A_4&= -t + \tau _4 H_{s_5}(A_5) \\ A_5&= -t + \tau _5 \end{aligned}$$

**Partial Derivative with Respect to** $\tau _3$:

$$\frac{\partial u}{\partial \tau _3}(t) = - H_{s_1}'(A_1) \cdot \tau _1 \cdot H_{s_2}'(A_2) \cdot \tau _2 \cdot H_{s_3}'(A_3) \cdot H_{s_4}(A_4) $$

**Sigmoid Derivative Expression:**

$$H_{s_i}'(x) = \frac{2}{s_i} \cdot \frac{e^{-2x/s_i}}{\left( 1 + e^{-2x/s_i}\right) ^2} $$

**Interpretation:** This full derivative captures both the **recursive nesting** and the **internal modulation** caused by $\tau _3$. The term in parentheses reflects the fact that $A_3$ depends nonlinearly on $\tau _3$ through both a direct term and an inner recursive function. If any $A_i$ is significantly off from zero, the corresponding derivative term vanishes. Hence, this structure explains how small misalignments lead to **complete signal cancellation**—mathematically modeling **ordinary forgetting** in weakly activated neural memory pathways.

### Case 2: High stress—overloading and distorted encoding

When the sensitivity (stress/sharpness/impressiveness) parameter $s_i \gg 1$, the sigmoid becomes flat and wide, making the recursive structure smoother and less distinct. Information diffuses across layers without sharp transitions.

**Recursive Structure (5-Layer Example):**

$$ u(t) = 1 - H_{s_1}\left( A_1\right) $$

$$\text {where} \quad \begin{aligned} A_1&= -t + \tau _1 H_{s_2}(A_2) \\ A_2&= -t + \tau _2 H_{s_3}(A_3) \\ A_3&= -t + \tau _3 H_{s_4}(A_4) \\ A_4&= -t + \tau _4 H_{s_5}(A_5) \\ A_5&= -t + \tau _5 \end{aligned} $$

**Partial Derivative with Respect to** $\tau _3$:

$$\frac{\partial u}{\partial \tau _3}(t) = - H_{s_1}'(A_1) \cdot \tau _1 \cdot H_{s_2}'(A_2) \cdot \tau _2 \cdot H_{s_3}'(A_3) \cdot H_{s_4}(A_4) $$

**Sigmoid Behavior under High Stress:**

$$ H_{S_i}'(x) = \frac{2}{s_i} \cdot \frac{e^{-2x/s_i}}{\left( 1 + e^{-2x/s_i}\right) ^2} $$

When $s_i \gg 1$, we have:

$$\begin{aligned} & H_{s_i}'(x) \approx \frac{0.5}{s_i} \quad \text {(broad, flat peak)}\\ & \quad \Rightarrow \quad \text {Signal spreads over time} \end{aligned}$$

**Interpretation:** In high- sensitivity (stress/sharpness/impressiveness) situations, the activation signal is smeared across time. Since the sigmoid is wide, the recursive structure becomes blurry, allowing cross-contamination of signals between layers.    

Even though the derivative is nonzero across a wide range, it lacks precision. As a result, memories may be partially encoded, temporally confused, or emotionally amplified. This behavior is linked to:

- *Trauma loops*: where signals persist across time due to broad sigmoid tails.
- *Overwriting*: where current events interfere with earlier layers.
- *Flashbacks or distorted temporal association*: caused by recursive diffusion.

**Boundary Behavior:** Conversely, as $s_i \rightarrow \infty$, the recursive sigmoid flattens, suppressing the formation of steep transitions and eliminating meaningful derivative activity. This mathematical degeneration corresponds to cognitive shutdown or trauma under extreme stress. Hence, this condition defines the opposite boundary of our recursive model—demonstrating how overstimulation structurally disables memory formation. It is not a speculative effect, but a mathematically inevitable outcome.

## From forgetting to focus: a recursive memory model

We model cognitive transitions through a deeply nested recursive function:

$$\begin{aligned} & u(t) = 1 - H_{s_1}\left( -t + \tau _1 H_{s_2}\left( -t + \tau _2 H_{s_3}\left( -t \right. \right. \right. \\ & \quad \left. \left. \left. + \tau _3 H_{s_4}\left( -t + \tau _4 H_{s_5}\left( -t + \tau _5 H_{s_6}(-t + \tau _6)\right) \right) \right) \right) \right) , \end{aligned}$$

where each $H_{s_i}$ is a smoothed Heaviside function parameterized by sensitivity (stress/sharpness/impressiveness) $s_i$.

When taking the partial derivative $\partial u / \partial \tau _2$, the result is nonzero:

$$ \frac{\partial u}{\partial \tau _2} = -\delta _{s_1}(-t + A_1) \cdot \tau _1 \cdot \delta _{s_2}(-t + \tau _2 H_{s_3}(\cdots )) \cdot H_{s_3}(\cdots ) \cdot \cdots , $$

while the other partial derivatives $\partial u / \partial \tau _i$ for $i = 3, 4, 5, 6$ vanish when $s_3 = s_4 = s_5 = s_6 = 0$, due to disjoint support of delta functions. This result models a state in which cognitive responses remain dormant until one specific threshold becomes active.

The model thus mirrors real-life experiences: we routinely forget or ignore most internal signals, until a sudden burst of awareness or attention emerges. This phenomenon resonates with the idea of interoceptive cognition and subconscious sensitivity, sometimes described as a “sixth sense” (Seth and Tsakiris 2014; Seth 2013). Similarly, layered activation can be seen in collective and emotional settings, as described by McDonnell (2014).

By embedding such cognitive selectivity in its recursive architecture, the function $u(t)$ mathematically encodes latent mental states that precede conscious attention.

### Applications across fields

The recursive mental potential model is not merely a mathematical construct—it holds interdisciplinary relevance with potential applications across diverse domains:

- *Neuroscience:* The model captures how cognitive signals propagate through hierarchical layers in the brain, offering a structured way to describe memory encoding, time-dependent recall, and the persistent imprint of trauma through sharp threshold activations.
- *Clinical Psychology:* It provides a new lens to interpret conditions such as PTSD, dissociation, and flashbacks. By modeling the interplay between stress sensitivity (sharpness $s_i$) and temporal thresholds ($\tau _i$), it elucidates how emotional events become deeply embedded or repressed.
- *Artificial Intelligence:* The framework suggests novel memory architectures where data is stored recursively, accessed via derivative operations, and capable of dynamic self-reference—enabling AI systems to simulate adaptive, experience-based awareness.
- *Philosophy of Mind:* The model bridges formal mathematics and metaphysical inquiry. It raises foundational questions about the nature of consciousness, the uniqueness of personal identity, and the plausibility of non-material continuity (e.g., reincarnation or soul), represented as recursive cognitive potentials.

### Building artificial recursive minds

AI systems inspired by the recursive Heaviside framework may incorporate:

- Layered temporal thresholds $\tau _i$ to represent sequential cognitive encoding across time or events,
- Sensitivity (stress/sharpness/impressiveness) parameters $$s_i $$ to modulate the intensity or salience of responses—analogous to emotional arousal or attentional filtering,
- Recursive partial derivatives to simulate introspection or meta-cognition—allowing the system to reference and modulate its own internal states.

## Conclusion and future directions

This paper introduced the *Recursive Heaviside Memory Architecture*, a mathematical framework for modeling cognition and memory as a layered, threshold-based system. Unlike linear memory timelines, our model captures the recursive, nonlinear accumulation of cognitive potential using smoothed step functions modulated by temporal thresholds $\tau _i$ and sharpness parameters $s_i$.

This formulation offers an explanation for various mental phenomena, including:

- Time-dependent recall and forgetting,
- Layered attention and recursive self-awareness,
- The irreversibility of memory processes—once a threshold is crossed, backward re-entry is blocked,
- Early warning signals (precursor derivatives) preceding conscious thought,
- Split or branching awareness paths, interpreted as cognitive multiverses.

The model connects directly to:

- *Neuroscience:* Describing hierarchical neural activation patterns and sharpness-modulated recall,
- *Clinical Psychology:* Providing formal insight into PTSD, trauma suppression, and dissociative memory,
- *Artificial Intelligence:* Offering a novel recursive architecture for adaptive, memory-based cognition,
- *Philosophy of Mind:* Grounding metaphysical ideas such as personal identity, soul, and irreversibility in a computable mathematical structure.

While the framework is theoretically rigorous and simulation-ready, several limitations remain. It requires further validation through experimental neuroscience and comparison with behavioral data. Moreover, translating recursive thresholds into biophysical correlates (e.g., neural firing patterns or memory circuits) remains an open question.

Nonetheless, this work provides a foundation for future interdisciplinary research across philosophy, psychology, and artificial intelligence. We hope it stimulates further exploration into the structure of memory, consciousness, and mind.

### Future directions

There remain several exciting directions for further research:

- *Mixed Derivatives:* Investigating how interactions between different layers—through cross-derivatives such as $\frac{\partial ^2 u}{\partial \tau _i \partial \tau _j}$, $\frac{\partial ^2 u}{\partial t \partial \tau _i}$, or $\frac{\partial ^2 u}{\partial s_i \partial \tau _j}$ —may explain phenomena such as reasoning, cognitive conflict, or associative memory pathways.
- *High-Order Derivatives:* Exploring second- or third-order derivatives of recursive potentials could shed light on long-term memory dynamics, decay profiles, and temporal fluctuations in emotional intensities.
- *Residual Dynamics and Dreams:* We think dreams arise from uncanceled residual terms—subtle leftover signals after most cognitive layers are satisfied. These residual components may provide a mathematical explanation for nocturnal cognition.
- *Application to Real Data:* We aim to validate this framework using neurophysiological data (e.g., EEG, fMRI), to investigate whether it can be mapped to observable brain activity patterns.

This work opens a novel path for integrating mathematics, cognitive science, and artificial intelligence. By viewing the mind as a recursive, threshold-sensitive system, we gain a clearer understanding of memory, awareness, and their inherent asymmetry, limitations, and creative potential.

## Data Availability

No datasets were generated or analysed during the current study.

## Appendix A: Full recursive structure of $u(t)$

Using sigmoid functions, the recursive potential can be written explicitly as:

$$ u_n(t) = 1 - \frac{1}{1 + \exp \left( -\dfrac{2(-t + \tau _n)}{s_n} \right) } $$

$$\vdots $$

$$ u_4(t) = 1 - \frac{1}{1 + \exp \left( -\frac{2}{s_4} \left( -t + \tau _4 \cdot \frac{1}{1 + \exp \left( -\frac{2}{s_5} \left( -t + \tau _5 \cdot \frac{1}{1 + \exp \left( -\frac{2}{s_6} \left( -t + \tau _6 \cdots \right) \right) } \right) \right) } \right) \right) } $$

$$ u_3(t) = 1 - \frac{1}{1 + \exp \left( -\frac{2}{s_3} \left( -t + \tau _3 \cdot \frac{1}{1 + \exp \left( -\frac{2}{s_4} \left( -t + \tau _4 \cdot \frac{1}{1 + \exp \left( -\frac{2}{s_5} \left( -t + \tau _5 \cdots \right) \right) } \right) \right) } \right) \right) } $$

$$ u_2(t) = 1 - \frac{1}{1 + \exp \left( -\frac{2}{s_2} \left( -t + \tau _2 \cdot \frac{1}{1 + \exp \left( -\frac{2}{s_3} \left( -t + \tau _3 \cdot \frac{1}{1 + \exp \left( -\frac{2}{s_4} \left( -t + \tau _4 \cdots \right) \right) } \right) \right) } \right) \right) } $$

A1 $$\begin{aligned} u_1(t) = 1 - \frac{1}{1 + \exp \left( -\frac{2}{s_1} \left( -t + \tau _1 \cdot \frac{1}{1 + \exp \left( -\frac{2}{s_2} \left( -t + \tau _2 \cdot \frac{1}{1 + \exp \left( -\frac{2}{s_3} \left( -t + \tau _3 \cdots \right) \right) } \right) \right) } \right) \right) } \end{aligned}$$

This formulation highlights the depth of recursion: each cognitive layer is gated by the activation of all deeper layers. The system forms a hierarchy of soft thresholds that depend both on time $t$ and the sequence of nested events $\tau _i$.

## Footnotes

1 This notion of recursive time layering is conceptually related to Kant’s idea of time as an inner intuition that structures all perception. See Kant (1929).
